# Cytomegalovirus Infection: An Underrated Target in Inflammatory Bowel Disease Treatment

**DOI:** 10.3390/jcm13010130

**Published:** 2023-12-26

**Authors:** Rossella Maresca, Simone Varca, Federica Di Vincenzo, Maria Elena Ainora, Irene Mignini, Alfredo Papa, Franco Scaldaferri, Antonio Gasbarrini, Maria Cristina Giustiniani, Maria Assunta Zocco, Lucrezia Laterza

**Affiliations:** 1 CEMAD Digestive Diseases Center, Fondazione Policlinico Universitario “A. Gemelli” IRCCS, Università Cattolica del Sacro Cuore, Largo A. Gemelli 8, 00168 Rome, Italy; rossella.maresca01@icatt.it (R.M.); simone.varca01@icatt.it (S.V.); federica.divincenzo01@icatt.it (F.D.V.); mariaelena.ainora@policlinicogemelli.it (M.E.A.); irene.mignini@guest.policlinicogemelli.it (I.M.); alfredo.papa@unicatt.it (A.P.); franco.scaldaferri@policlinicogemelli.it (F.S.); antonio.gasbarrini@unicatt.it (A.G.); lucrezia.laterza@policlinicogemelli.it (L.L.); 2 Dipartimento Universitario di Medicina e Chirurgia Traslazionale, Università Cattolica del Sacro Cuore, 00168 Rome, Italy; 3 Department of Pathology, Fondazione Policlinico Universitario “A. Gemelli” IRCCS, Largo A. Gemelli 8, 00168 Rome, Italy; mariacristina.giustiniani@policlinicogemelli.it

**Keywords:** infections, CMV, IBD, Crohn’s, ulcerative colitis

## Abstract

CMV infection is still a matter of concern in IBD patients, especially regarding the disease’s relapse management. Why IBD patients, particularly those affected by ulcerative colitis, are more susceptible to CMV reactivation is not totally explained, although a weakened immune system could be the reason. Various techniques, ranging from serology to histology, can be employed to detect intestinal CMV infection; however, there is currently disagreement in the literature regarding the most effective diagnostic test. Furthermore, CMV involvement in steroid resistance has been broadly discussed, but whether CMV infection is a cause or consequence of the disease severity and, consequently, steroid refractoriness is still debated. Its potential contribution to the lack of response to advanced therapy and small molecules must be more valued and wholly explored. In this review, we look at the actual literature on CMV in IBD patients, and we suggest a pragmatic algorithm for clinical practice management of CMV infection.

## 1. CMV Incidence and Prevalence in IBD Patients: The Size of the Problem

Cytomegalovirus (CMV) is a member of the herpes-virus family, which causes a broad spectrum of human illnesses depending on the host, but most commonly an asymptomatic viral infection (only detectable by serology or viral DNA). Clinically apparent infections (CMV disease) usually present as a mononucleosis-like syndrome but can affect potentially any organ of the human body [1,2].

The epidemiology of CMV infection and colitis varies according to the definition used to diagnose the infection due to the lack of a gold standard definition of clinically relevant CMV infection and CMV intestinal disease, to the severity of colitis, to the studied population, including immunological status, and to the geographical distribution. The highest prevalence of CMV disease is found in studies that used the positive serum polymerase chain reaction (PCR), followed by studies using antigenemia, where the definition of CMV infection is serum CMV replication.

The proportion of individuals with evidence of prior exposure to CMV varies worldwide, with seroprevalence rates in the adult population ranging between 40% in highly industrialized areas and 100% in developing countries [3]. Additionally, some studies indicate that the prevalence of CMV-specific antibodies rises with age, with rates ranging from 47% in children aged 10–12 to 81% in young adults aged 36–60. It also differs based on race, ethnicity, and other variables, such as female sex, foreign birthplace, low family income, overcrowding in the family, and low family education [4,5].

Regarding patients affected by inflammatory bowel disease (IBD), a recent meta-analysis showed no difference in latent CMV infection between IBD and healthy controls (69.6% vs. 51.8%, odds ratio [OR] = 1.36, 95% confidence interval [CI] = 0.45–4.14, *p* = 0.59). However, the authors found a significant difference between the two groups regarding the CMV IgM (5.5% vs. 0.59%, OR 7.14, 95% CI = 1.58–32.25, *p* = 0.01), with higher rates among IBD patients. Similarly, there was a significantly increased risk in IBD patients of serum CMV replication, as evidenced by CMV antigenemia (40.4% vs. 6.6%, OR 7.4) and the level of blood CMV DNA (42.5% vs. 26.4%, OR 4.99) [6,7]. These data agree with previous studies showing that subclinical reactivation of CMV during immunomodulator, steroid or biological therapy is common in IBD patients, but it is nearly always asymptomatic and self-limiting even if therapy is continued [7,8,9].

There are very few data on CMV detection in stool, and those available focus on IBD patient cohorts. However, a Scandinavian study in a cohort of IBD patients with active UC (*n* = 75) using quantitative stool-PCR found a prevalence of CMV of 40.4% compared to 21.3% determined via tissue immune histochemistry (IHC) [10].

When considering intestinal CMV infection, the association between IBD and CMV colitis has been recognized for 50 years [11]. However, data on the incidence and prevalence of CMV colitis among IBD patients still need to be clarified, mainly due to selection bias and heterogeneity in the methods used to diagnose CMV infection. The highest prevalence of CMV infection has been seen in studies that used tissue PCR greater than 10 copies/mg tissue. In an observational study, the CMV genome was detected using PCR on intestinal tissue samples in 32.9% of the IBD patients and only 2.4% of the controls, patients who underwent colonoscopy for other reasons such as anemia or non-IBD [12]. Studies regarding patients with inactive or mild-to-moderate ulcerative colitis (UC) have not shown an increased risk of CMV colitis, as diagnosed with hematoxylin and eosin (H&E) and IHC. Most of the literature, however, focuses on severe and/or steroid-refractory UC, terms used interchangeably and often not clearly defined. Therefore, the results are difficult to interpret [13].

The prevalence of CMV colitis diagnosed with H&E or IHC varies considerably between UC patients [14,15], ranging from 4.5% in new onset UC [16] to 16.6% in acute severe UC [17,18], and up to 36% in patients with acute severe colitis resistant to intravenous steroids [19]. The highest values were found in patients who needed an urgent colectomy, where the prevalence ranged between 11.5% and 27% [13].

Interestingly, a large observational study by Bontà et al. revealed a prevalence of 1.37% of CMV disease diagnosed with IHC in a cohort of 1023 consecutive IBD patients. All the patients who developed CMV colitis were receiving immunosuppressive medication, such as steroids, azathioprine, or infliximab, most frequently in combination, or had HIV infection (*n* = 1). None of the diagnosed cases was treated with infliximab as monotherapy [20]. Similarly, other research indicates that anti-TNFalpha has no effect on the CMV infection’s clinical course [21,22]. In particular, a previous prospective study found that infliximab was not associated with progression from CMV infection to disease [22]. Additionally, in another study, the authors even suggested treating colitis flare-ups associated with CMV reactivation with infliximab or adalimumab [21].

Contrary to UC, most studies have not shown an increased prevalence of CMV colitis diagnosed via IHC in patients with Crohn’s disease (CD). Some theories have been proposed to explain the different prevalence of CMV colitis among CD and UC patients. However, these results were obtained from small studies and should be carefully interpreted. One explanation suggests that in patients with IBD, tumor necrosis factor-alpha (TNF-α) might be associated with CMV infection or reactivation. At the same time, interferon-γ (IFNγ), produced by CD4+ T cells, might suppress CMV reactivation. According to some authors, in CD, the Th1-type inflammatory process could be enhanced with high expression of IFNγ, thereby explaining the lower prevalence of CMV colitis [13].

Factors that have been associated in previous studies with an increased risk of CMV reactivation in IBD patients include the female sex, pancolitis, advanced age (age > 30 years) [23,24,25], immunosuppressive therapy (steroids and azathioprine) [23] (OR 6.7), disease duration less than 60 months [OR 7.7], and blood leukocytes count less than 11/nl (OR 4.49) [23].

## 2. Clinical Implications

### 2.1. Clinical Presentation

Primary CMV infection is frequently asymptomatic but sometimes may present as a mononucleosis-like syndrome accompanied by systemic symptoms such as fever, leucopenia, splenomegaly, and lymphadenopathy [26]. Moreover, although uncommon, involvement of other organs is possible even in patients with IBD [27]. CMV colitis occurs with symptoms such as increased stool frequency, rectal bleeding, and tenesmus. For this reason, it may be difficult to distinguish it from an underlying flare of disease [26]. However, IBD patients seem to be more vulnerable to CMV reactivation, especially those with UC, probably because of an impaired immune reaction to the virus [28].

Following the initial phase of infection, CMV can move into a latent state in a variety of cell types, including cells of colonic mucosa. During this time, the immune system, in particular natural killer (NK) cells, maintains a control on the virus.

In IBD patients, there is an impaired immune system (NK and T cells) and an increase in inflammatory cytokines, especially TNF-α, which would encourage CMV reactivation. Indeed, TNF-alpha could trigger infected monocytes to differentiate into macrophages, which would enable the reactivated CMV to infect surrounding cells (interstitial, vascular endothelial, and epithelial cells) [29]. This would increase the production of IL-6 and trigger a vicious cycle with worsening colitis [30].

### 2.2. CMV Infection, Steroid Resistance and Outcomes

To date, differentiating a UC relapse from CMV colitis represents a major challenge in clinical practice. Furthermore, the clinical implications of CMV colitis, such as possible steroid refractoriness, must be evaluated with a view to treatment.

Regarding the correlation between the endoscopic severity of UC and CMV, there are contradictory results. While some research indicates that patients with CMV antigenemia show a higher prevalence of endoscopically severe UC than patients without antigenemia (*p* = 0.016) [31], other studies did not find a statistically significant association between the Mayo endoscopic score and the detection of CMV DNA in intestinal tissue [32]. Furthermore, specific endoscopic findings in patients with UC complicated by CMV infection were identified, such as pounced-out ulcers, wide mucosal defects and irregular ulcerations. In a study, the sensitivity of irregular ulceration for positive CMV was 100%; meanwhile, mucosal defects showed a specificity of 95% [33]. Similarly, a higher proportion of punched-out ulcers were observed among the patients with CMV colitis compared to the non-CMV control group (52.0% versus 20.0%, *p*-value = 0.04), with a sensitivity of 52.0% and a specificity of 77% [34].

However, Iida et al. revealed that punched-out ulcers were among the colonoscopic findings that were similar in CMV (+) and CMV (−) individuals [35]. Importantly, steroid-refractory illness had a much higher prevalence of these ulcers. These results point away from CMV infection and toward the possibility that perforated ulceration is a sign of disease severity, specifically of steroid refractoriness.

An increasing amount of evidence supports the notion that patients with acute severe colitis and CMV infection are more resistant to corticosteroid treatment than individuals without the infection.

Specifically, several meta-analyses suggest that CMV infection in IBD patients is associated with a two- to four-fold higher risk of steroid resistance [6,36,37].

Similarly, in a pediatric multicenter retrospective study [38], the authors demonstrated that, compared to CMV-negative patients, a higher proportion of CMV (+) patients were resistant to intravenous corticosteroid administration (*p* = 0.009).

Nevertheless, it is still completely unknown what leads to steroid resistance in IBD patients during CMV infection and if CMV infection is a cause or consequence of the disease severity and steroid refractoriness. A recent work by Wang et al. attempted to explain this mechanism [39]. It is commonly known that glucocorticoids, when bound to glucocorticoid α (GRα) receptors, have anti-inflammatory and immunosuppressive properties; conversely, glucocorticoid β (GRβ) binding has no effect. As demonstrated, the GRβ/α ratio significantly increases during the lytic phase of CMV reactivation.

Consequently, the refractory response to steroid treatment may be explained by these changes in receptors [28].

However, the influence of CMV on the response to therapies other than steroids, including the most recent biological and advanced one, has been poorly investigated.

Moreover, several studies have shown worse long-term outcomes in patients with CMV infection [40,41,42].

In a retrospective study, Zagorowicz et al. found that UC patients with CMV infection and high counts of CMV-positive cells on biopsy (defined as ≥5 CMV IHC-positive cells per section) had significantly lower colectomy-free survival than patients with less or no IHC-positive cells (1.9 vs. 3.2 years, respectively, *p* = 0.014) [40]. Schenk et al. found similar results in patients whose colonic biopsies revealed CMV-DNA positivity [41]. In fact, they reported that, over a median follow-up of nearly four years (52 months), patients with positive detection of CMV-DNA in biopsy had a higher rate of proctocolectomy (33.3% vs. 11.9%, *p* < 0.005). In order to assess the outcomes of CMV infection, a recent study examined 254,839 pediatric hospitalizations connected to IBD, including both CD and UC. As a result, they observed that CMV infection was associated with 3.5 times higher odds of in-hospital mortality (OR: 3.58; CI: 1.85 to 6.93, *p* < 0.001) and severe IBD (OR: 3.31; CI: 2.54 to 4.32, *p* < 0.001), which in turn contributed to a longer hospital stay (in fact, CMV infection increased the length of stay by 9 days) [42].

On the contrary, Delvicourt et al., in a case-control (*n* = 26) retrospective study, found no differences in terms of the length of hospital stay and 3-month colectomy rate [43]. They attempted to evaluate the impact of CMV reactivation on hospitalized patients experiencing an IBD flare and the effect of antiviral therapy on IBD flare. They found 8.7 days vs. 9.7 days (*p* = 0.42) and a colectomy rate of 15.4% vs. 23.1% (*p* = 0.48) for CMV+ and CMV patients, respectively. Similar results were obtained from a small pediatric case-control study where CMV positivity was not statistically associated with an increased risk of colectomy at 12 months (*p* = 0.429) [44].

## 3. Methods for Diagnosis of CMV Infection

The diagnostic challenge in this complex clinical picture is to distinguish CMV colitis from IBD exacerbations, as both conditions can present with similar clinical features. Serology, histology, and PCR for CMV DNA in the blood, stool or intestinal tissue are the techniques that can be used to identify intestinal CMV infection.

### 3.1. Serology

Serological diagnosis of CMV infection relies on detecting specific antibodies the host produces in response to the virus. IgM serology has a sensitivity of between 15 and 60% in detecting CMV in a 2004 study of a cohort of 64 IBD patients, including 10 with CMV infection [24]. Enzyme-Linked Immunosorbent Assay (ELISA) is the most common serological method used to detect CMV antibodies. In ELISA, viral antigens are immobilized on a solid phase, and patient serum is applied. If specific anti-CMV antibodies are present, they bind to the antigens. Subsequent enzymatic reactions generate a color change, which is quantified to determine the antibody levels [45].

Another essential serological technique is the Immunofluorescence Assay (IFA), which uses fluorescent antibodies to detect CMV-specific immunoglobulins in patient samples [46]. This method is beneficial for confirming positive ELISA results and distinguishing between IgM and IgG antibodies.

Blood serology for CMV has limited diagnostic value for CMV colitis due to the high adult seroprevalence of the virus, as previously mentioned [47].

Therefore, serology has limited utility in diagnosing CMV reactivation in individuals with IBD, only allowing doctors to rule out CMV infection in patients who are negative for anti-CMV antibodies [48].

### 3.2. Mucosal Biopsies: Immunohistochemistry and PCR

Histological examination of colonic mucosa biopsies is a critical tool for diagnosing CMV infection in IBD patients. Histological assessment of CMV infection in colonic mucosa biopsies involves the examination of tissue samples obtained via endoscopy or surgical procedures [49]. Quality colonoscopy is essential for the diagnostic algorithm of patients with suspected CMV infection, and it must be performed under conditions of adequate bowel preparation. This can be achieved either using oral solutions or using enemas, especially in cases of severe acute ulcerative colitis where rectoscopy allows us to perform sampling for CMV search and rapid endoscopic Mayo score evaluation [50]. The characteristic features of CMV infection in these samples include cytomegalic cells, intranuclear inclusions, and characteristic nuclear and cytoplasmic inclusions [49].

Cytomegalic cells are enlarged cells with characteristic nuclear and cytoplasmic changes. These cells often have large, basophilic intranuclear inclusions indicative of CMV infection. In the cytoplasm, there may be granular or eosinophilic inclusions. The presence of these cytomegalic cells in colonic mucosa biopsies is highly suggestive of CMV infection [51].

Several staining methods can be used to confirm the diagnosis. H&E staining is a standard histological method highlighting the characteristic cellular changes [52]. However, IHC can be even more specific, using antibodies against CMV antigens to identify the virus within infected cells. High specificity [92–100%] can be achieved with H&E staining, while the sensitivity ranges from 10% to 87% [53].

In 2013, Mills et al. showed that CMV-PCR on formalin-fixed, paraffin-embedded (FFPE) tissue gastrointestinal biopsies complements IHC [54]. In fact, in a total of 102 FFPE gastrointestinal biopsy specimens from 74 patients, CMV DNA was detected via PCR in 90.9% of IHC-positive and 14.5% of IHC-negative tissues. Meanwhile, Kandiel et al. have demonstrated that staining the colonic CMV histology with a particular IHC that is specific to one of the immediate early antigens increases the diagnostic sensitivity [78–93%] and specificity (92–100%) of the examination [27].

Kim et al. identified active gastrointestinal (GI) CMV illness, defined as histological detection of intranuclear inclusion bodies or positive IHC and clinical improvement on ganciclovir treatment, using both interferon γ-releasing assays [IGRA] for CMV and CMV PCR. For predicting gastrointestinal CMV illness, the sensitivity and specificity of the CMV replication in biopsy tissue (positive IHC staining) and low CMV IGRA results were 92% (95% CI = 62–100) and 100% (95% CI = 74–100), respectively [15].

Either way, PCR on GI biopsy tissue is the diagnostic method recommended by the European Crohn’s and Colitis Organization (ECCO) for diagnosing CMV colitis in IBD patients [55].

Real-time PCR increases the sensitivity to identify CMV in FFPE tissue of GI biopsies, as demonstrated by McCoy et al. [47]. In their study, qPCR analysis showed positive results in 88 out of the 91 CMV-positive samples that were histologically confirmed, obtaining a sensitivity of 96.7%. In addition, 78 out of the 79 negative controls tested negative via qPCR, yielding a specificity of 98.7%.

Furthermore, in a study published in 2015, Zidar et al. compared immunohistochemistry and qPCR in resected bowel samples from 12 IBD patients [56]. Tissue samples were obtained from different sites of the resected colonic samples. The highest densities of CMV-positive cells were found in samples from the base of ulcers or the edge of ulcers, regardless of the test used and underling the fact that the number of sampled biopsies and/or the number of investigated levels is more important than the choice of diagnostic method [56].

Distinguishing CMV infection from other inflammatory changes in IBD is crucial. Patients with IBD often exhibit histological signs of inflammation in the colon, such as increased numbers of inflammatory cells, architectural distortion, and crypt abscesses. Therefore, these inflammatory changes in colonic mucosa biopsies must be carefully evaluated in the context of CMV diagnosis. A comprehensive assessment should consider the overall clinical presentation and the results of other diagnostic tests, such as PCR-based assays for CMV DNA in blood or tissue.

### 3.3. Serum and Fecal PCR

PCR-based assays can also detect CMV DNA in blood or other body fluids, providing direct evidence of active viral replication. This approach is highly sensitive and is often used with serological tests for a comprehensive diagnosis.

A prospective study was conducted in 2023 on 117 patients with clinical suspicion of CMV colitis. Compared to colonoscopy and histology, plasma CMV-PCR had a 94.7% specificity and a 66.7% sensitivity [57].

However, according to the ECCO statement regarding IBD patients, this high sensitivity of the PCR assay may lead to inadequate specificity when diagnosing an active CMV infection since it may be able to identify tiny amounts of CMV that are not harmful to the colonic mucosa or the latent form of the virus [58]. To get around this drawback, PCR primers that identify CMV in its reactivated state, but not in its latent form, must be chosen.

A pilot study conducted in 2010 investigated the use of stool PCR in 21 patients with IBD flare-ups unresponsive to steroids. It demonstrated that in comparison to PCR-based CMV detection in mucosal biopsies, the sensitivity, specificity, and accuracy of the stool test for CMV DNA detection were 83, 93, and 90%, respectively [59].

Ganzenmueller et al. examined 66 patients’ lower intestinal tract biopsies and fecal samples using quantitative CMV PCR. They showed that stool PCR had a good specificity of 96% but a poor sensitivity (67%) for diagnosing CMV intestinal disease [60].

This study highlights how fecal PCR analysis can be a useful diagnostic tool for CMV intestinal disease by avoiding an endoscopic examination for a positive result, even though it does not help rule out CMV infection in case of a negative result [60]. In contrast, a more recent study in 2020 that included patients with ulcerative colitis revealed that the stool-CMV PCR had a sensitivity of 84.7% and a specificity of 71.4% [10].

In conclusion, more studies regarding PCR on stool still need to be conducted to determine the true place of this test in the diagnostic algorithm of CMV-related colitis.

**Figure 1:**
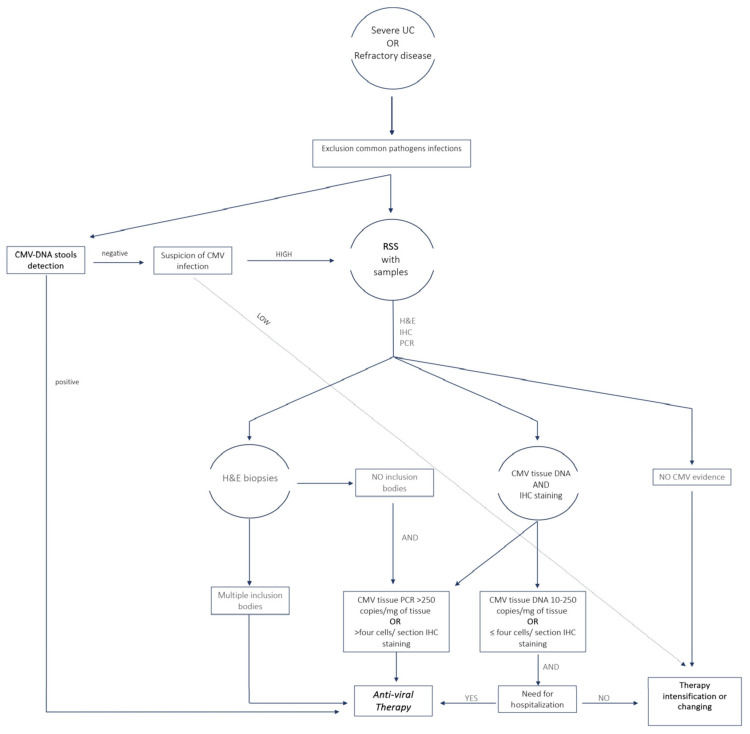
Algorithm for the diagnosis of CMV infection and selection of patients to treat for clinical practice. UC, ulcerative colitis; H&E, hematoxylin and eosin; IHC, immune histochemistry; PCR, polymerase chain reaction; RSS, rectosigmoidoscopy.

The sensitivity, specificity and strengths of the main diagnostic methods are shown in Table 1. In Figure 1 we propose an algorithm that from the diagnosis can direct physicians in selecting patients for treatment.

## 4. Treatment

### 4.1. When to Treat? Indications for Specific Antiviral Therapy

The development of CMV colitis is associated with an increased risk of poor outcomes, including toxic megacolon, colectomy, need for rescue therapy and increased rates of disease flares [38,41,61,62,63,64]. Therefore, it is crucial to maintain a high level of clinical suspicion of CMV infection/reactivation in UC patients presenting with a worsening of their gastrointestinal symptoms, regardless of their immunosuppression status. A delay in diagnosis and subsequent management may be associated with unfavorable outcomes, including increased colectomy rates.

Previous studies showed that in steroid-dependent or steroid-refractory UC patients, those receiving antiviral treatment experienced a significantly higher clinical remission rate at 12 months compared to those who did not receive such treatment [65].

It is interesting to note that conflicting findings about the risk of colectomy following antiviral therapy for CMV were found in two separate meta-analyses involving 176 and 333 UC patients with CMV, respectively [66,67]. Indeed, the first study found that patients who had received antiviral therapy had a higher risk of a 30-day colectomy [66]. In contrast, the second study found that antiviral therapy may be helpful concerning he colectomy risk in a subgroup of UC patients refractory to corticosteroids (OR, 0.20; 95% CI = 0.08–0.49) [67]. Furthermore, in these patients, the authors observed a lower rate of colectomy in those for whom the diagnosis was made using histological criteria (H&E and/or IHC) rather than with tissue PCR [67]. To some extent, the discordance of these results may derive from differences in the CMV burden and in the methods used to perform the diagnosis in each study.

Thus, antiviral therapy may be required by only a subset of IBD patients with CMV disease. However, to date, only limited information is available in the literature concerning the relationship between the tissue viral load, measured by viral inclusion in IHC [40,68] or CMV-DNA copies [32], and the UC outcome.

Jones A. et al. showed that patients with low-grade CMV colitis (<5 viral inclusions on IHC) were significantly more likely to undergo surgery than those with high-grade colitis (HR 2.13, 95% CI = 0.85–5.33). Interestingly, compared to patients with high-grade colitis who received treatment, patients with low-grade CMV colitis who did not receive anti-viral therapy had a nearly five-fold higher likelihood of undergoing surgery within a year (HR 4.81, 95% CI = 1.60–14.48). However, Nguyen et al. demonstrated that anti-viral treatment did not change the colectomy rates in UC patients with low-grade disease but did change the outcomes in those with high-grade disease (44% if treated and 83% if untreated) [69].

Similarly, Roblin et al. revealed that most patients with a higher colonic viral load (CMV DNA load above 250 copies/mg of tissue) responded to anti-viral therapy despite failing three consecutive lines of immunosuppressant therapy [32]. However, quantitative RT-PCR is only sometimes available in clinical settings; hence, clinicians often assess CMV DNA-positivity only qualitatively.

In a recent retrospective case-control study, Wang et al. showed that in UC patients with positive colonic CMV virocytes diagnosed via H&E and/or IHC, the anti-viral therapy significantly improved the surgery-free survival within 30 days, with a difference sustained for 70 months [70]. Okahura et al. demonstrated that patients with a high tissue viral load of CMV may respond to anti-viral treatment alone without additional therapy for UC, whereas patients with a low viral load (<5500 copies/μg DNA) might benefit from intensified UC therapy [25]. On the other side, a small study involving 20 patients suggested that the absence of endoscopically visible large ulcers (>5 mm) in patients with active UC and a positive CMV DNA via mucosal PCR assay are signs of a latent CMV infection, therefore not requiring an anti-viral therapy and likely to respond to conventional immunosuppressive therapy [71]. Conversely, patients with large ulcers poorly responded even when they were given the combined therapy [71].

Whether CMV reactivation exacerbates the progression of IBD is still unknown. However, in patients with low CMV burdens, the underlying IBD colitis is likely the primary cause of gut inflammation, and CMV is just “an innocent bystander”. On the other hand, in patients with high CMV burdens, the virus appears to be an active pathogen; in these cases, UC-induced inflammation may not have a principal impact on gut inflammation.

Hence, despite the lack of controlled trial data, there are sufficient pieces of evidence to support anti-viral therapy only in patients with steroid-dependent/refractory moderate-to-severe colitis and high-grade colonic CMV reactivation diagnosed via H&E staining with IHC and/or CMV tissue PCR (CMV DNA load above 250 copies/mg of tissue [32] or ≥five viral inclusions evident on IHC in each biopsy specimen) [40,68]. However, while some algorithms have been proposed [72,73], an exact threshold to determine which patients might benefit from anti-viral therapy is still to be defined.

Pillet and colleagues presented a treatment algorithm that categorized patients according to the density of CMV in colon samples: individuals with high-grade CMV density, individuals with low-grade CMV density, or individuals without CMV. Specifically, they stated that patients with high-grade CMV (defined as CMV tissue DNA > 250 copies/mg or >four inclusions on IHC) would require anti-viral and anti-TNF therapy; meanwhile, patients with low-grade CMV (CMV tissue DNA 10–250 copies/mg or ≤4 inclusions on IHC) would need only anti-viral therapy in case of severe disease (defined as large colic ulcers [>5 mm] at colonoscopy or need to hospitalization).

On the other hand, individuals lacking CMV would need to treat the underlying UC with a more intense immunosuppressive regime [72].

Recently, Mourad et al., in a comprehensive review, suggested the administration of anti-viral treatment in two circumstances: firstly, in patients with acute severe colitis and multiple inclusion bodies on colonic samples, regardless of the CMV tissue PCR or positive IHC strains; and secondly, in patients with negative inclusion bodies but high CMV tissue PCR (>250 copies/mg of tissue) or high IHC staining (>four cells/section) [28].

### 4.2. Comparison of Guidelines: IBD Guidelines vs. Infectious Disease Guidelines

The remission rate of UC patients after antiviral therapy for CMV colitis is high (67% to 100%) [17,19,74]. The preferred antiviral agent for treating CMV colitis is intravenous ganciclovir at 5–7.5 mg/kg twice daily for two weeks [53,75,76]. Due to the drug’s renal excretion, the dosage and frequency should be adjusted in patients with renal dysfunction [77], and strict monitoring of patients’ renal function and electrolytes during the treatment is recommended [78].

Prolonged intravenous antiviral therapy (2–3 weeks) usually requires hospitalization [79]. However, ganciclovir may be replaced with oral valganciclovir (1 g 3 times daily) in those treated as outpatients.

Despite low-quality evidence, the British Society guidelines recommend for hospitalized patients the intravenous administration of ganciclovir (5 mg/kg twice daily) while maintaining conventional therapy with corticosteroids or rescue medications with infliximab or cyclosporine [80].

The 2014 ECCO guidelines recommended the administration of intravenous ganciclovir (5 mg/kg twice daily) for 3–5 days. Depending on the clinical course and expert advice, switching to oral valganciclovir (900 mg twice daily) for the remaining 2–3 weeks could be considered [55].

More recently, however, the ECCO has extended the period of intravenous ganciclovir 5 mg/kg twice daily to 5–10 days, with the possibility of an earlier switch to oral therapy depending on clinical response [78].

Foscarnet, administrated intravenously (90 mg/kg) twice daily for 2–3 weeks, has been proposed as an alternative treatment in cases of resistance or intolerance (e.g., myelotoxicity) to ganciclovir. In case of severe adverse events, such as severe neutropenia and thrombocytopenia, consulting an infectious disease specialist is strongly recommended [55,78].

In rare cases of systemic CMV reactivation causing meningoencephalitis, pneumonitis, hepatitis, or esophagitis, all the guidelines recommend immediate discontinuation of the immunosuppressive agents and prompt treatment of the CMV infection with antivirals according to the indications of infectious disease specialists, since this regimen is associated with clinical improvement and decreased mortality [55,78,80]

### 4.3. Implications of Immunosuppressant and Advanced Therapies

Immunosuppressive conditions are known to frequently result in CMV infection or reactivation. In IBD, the use of immunosuppressants and treatment with biologics may result in an alteration in the immune system, thus favoring CMV reactivation. While the British Society guidelines recommend to continue conventional immunosuppressive therapy during antiviral therapy [80], the ECCO guidelines suggest considering the cessation of all immunomodulatory therapies, including steroids, until the CMV symptoms are controlled (evidence level 5, experts’ opinion) [55].

Several therapeutic schedules have been proposed for the treatment of steroid-dependent/resistant colitis with CMV reactivation, including a rapid steroid tapering and discontinuation [38,81,82].

On the other hand, previous studies suggested that any already initiated IBD treatment, including conventional steroid therapy, should be continued during antiviral therapy for CMV colitis, and medical rescue therapy should be prescribed when necessary [76,77]. Accordingly, a retrospective multicenter study involving 110 hospitalized patients with refractory UC, associated with CMV disease, demonstrated that administration of infliximab or cyclosporine together with ganciclovir did not cause a worse outcome (colectomy rate at 1, 3 and 12 months) compared with ganciclovir alone [83].

It has been demonstrated that thiopurines interfere with the function of natural killer cells and T lymphocytes specific to CMV. Shukla et al. recently conducted a meta-analysis that included 16 observational studies and found that exposure to thiopurines was associated with a higher risk of CMV reactivation (1273 patients, OR 1.56, 95% CI = 1.01–2.39) [84]. When the analysis was restricted to hospitalized patients, the thiopurine results remained consistent. In addition, there was no significant link between exposure to TNF-alpha antagonists and risk of CMV reactivation (OR 1.44, 95% CI = 0.93–2.24) [84].

Similarly, Pillet et al. try to assess the relationship between CMV colitis and UC therapy with anti-TNF-alpha (adalimumab or infliximab) or azathioprine (AZA) in 109 disease flare-ups (anti-TNF-alpha = 69 and AZA = 40) [21]. The percentage of CMV reactivation was similar in the two groups (35% vs. 38% in patients receiving anti-TNF and AZA, respectively). In a sub-analysis of patients who required optimization of the anti-TNF therapy, the clinical response (defined as partial Mayo score < 3) was not significantly different in patients exhibiting or not a detectable CMV DNA load in colonic tissue just before optimization (*p* = 0.52) [21]. Since the authors found no evidence of a reciprocal negative relationship between anti-TNF medications and CMV infection, their findings imply that these medications may be used to treat flare-ups brought on by CMV reactivation.

Accordingly, the study by Lavagna et al. showed that administration of infliximab in CMV-seropositive patients with refractory CD did not cause the reactivation of CMV, since no patient was positive on blood CMV PCR during the three sessions of infliximab therapy [7].

Treatment with anti-TNF alpha does not adversely affect the outcome of patients with CMV reactivation [21,85].

Moreover, previous studies suggested that by lowering the TNF-α levels and the macrophage differentiation in the colon tissue, anti-TNF agents inhibit CMV reactivation and reduce the incidence of CMV colitis, thereby being preferable to other immunosuppressant therapies used to treat CMV reactivation-associated flare-ups in UC patients [79].

Finally, in a multicenter retrospective study, the authors found no additional risk of colectomy in CMV-positive patients associated with exposure to infliximab (IFX) or cyclosporine [83].

Cyclosporine A (CyA) selectively suppresses T lymphocyte-mediated immune responses by altering the calcineurin–calmodulin interaction. A small Japanese study examines the effects of an ongoing CMV infection after treating steroid-refractory UC with Cya [86]. They included 23 patients treated with intravenous CyA. None of the patients had an active CMV infection prior to receiving CyA treatment. Out of the 23 UC patients treated with CyA, 18 developed a CMV infection. Following CyA treatment, the CMV infection started to manifest, on average, 8.5 days later (range: 4–20 days). Uncontrolled, severe, deteriorating colitis necessitated surgical treatment in 15 out of the 18 patients (83.3%) with CMV infection. Of the 18 patients with CMV infection, 15 (83.3%) required surgery due to severe and rapidly worsening colitis.

Vedolizumab is an anti-α4β7 integrinmonoclonal antibody that inhibits leucocyte trafficking in the gut. Typically, it has very limited systemic effects [87].

Even though several works have reported the safety of vedolizumab and focused on its risk of infection [88], a recent case report described a case of CMV colitis in a patient undergoing monotherapy with the anti-integrin, indicating that it might be a risk factor for UC CMV colitis. In addition, vedolizumab may even be useful in treating CMV colitis as showed in two case reports [89].

The available data on the other advanced therapies are still scarce.

Only two cases of CMV colitis in patients receiving ustekinumab as maintenance were reported in the UNIFI study [90]. To the best of our knowledge, the OCTAVE Induction 2 study contains the only information on small molecules. In this study, a single patient receiving tofacitinib 10 mg twice daily developed CMV colitis [91].

Actually, despite the theoretical risk of worsening the outcome of CMV colitis, immunosuppressants are maintained in most of the cases for the control of disease activity [1,20,38,43,62,81,83,92,93,94,95,96] since, especially in patients with low viral loads and a low number of IHC-positive cells in the colon, CMV clearance may be parallel to the achievement of remission induced by immunosuppressants, even in patients who did not receive antivirals [93].

Therefore, the correct therapeutic schedule for corticosteroid and immunosuppressive agents during CMV reactivation in refractory IBD is still to be determined. Additional studies are required to explore the effects of immunomodulators in the treatment of IBD complicated by CMV colitis.

Most evidence suggests direct rectosigmoidoscopy with biopsy in patients at high risk of CMV. However, considering the non-invasiveness, relatively low cost and diagnostic accuracy, a suggested alternative approach, particularly for outpatients, might be first to determine the presence of CMV-DNA in the stools and to only perform RSS in negative patients with a high suspicion of CMV infection. There is a no universally accepted cut-off for defining PCR positivity in stools.

Further studies are needed to confirm the diagnostic accuracy and to define the correct cut-off and the cost-effectiveness of this strategy in a clinical setting.

See the text for more details.

## 5. Conclusions

CMV colitis could have a major impact on the IBD course, particularly during UC flares. Even if its role in reducing the clinical response during steroid therapy is well established, practical approaches to the infection largely vary in clinical practice due to the different implemented diagnostic methods, with consequent variable diagnostic accuracy and different thresholds for treatment reported in the literature. Furthermore, the role of CMV colitis as a possible determinant of a lack of response to new biological drugs and small molecules has not been systematically investigated in the literature, thus it could be largely underestimated. Further studies are required to investigate the potential role of CMV during new advanced therapies and its optimal management in this setting to improve response to therapy.

## Figures and Tables

**Table 1:** Sensitivity, specificity and accuracy of diagnostic methods.

Test	Sens	Spec	Pro
Serology	15–60%	96–99%	Easily accessible, fast
Serum PCR	66.7%	94.7%	Easily accessible, fast
Histology w/IHC	78–93%	92–100%	Proves intestinal disease
Tissue PCR	96.7%	98.7%	Proves intestinal disease
Fecal PCR	83–96%	67–93%	Less invasive

PCR, polymerase chain reaction; IHC, immune histochemistry.

## Data Availability

Not applicable.
